# Hydrochar from Pine Needles as a Green Alternative for Catalytic Electrodes in Energy Applications

**DOI:** 10.3390/molecules29143286

**Published:** 2024-07-11

**Authors:** Assunta Marrocchi, Elisa Cerza, Suhas Chandrasekaran, Emanuela Sgreccia, Saulius Kaciulis, Luigi Vaccaro, Suanto Syahputra, Florence Vacandio, Philippe Knauth, Maria Luisa Di Vona

**Affiliations:** 1 Department of Chemistry, Biology and Biotechnology, University of Perugia, Via Elce di Sotto 8, 06123 Perugia, Italy; elisa.cerza@dottorandi.unipg.it (E.C.); luigi.vaccaro@unipg.it (L.V.); 2 Tor Vergata University of Rome, Department Industrial Engineering and International Laboratory: Ionomer Materials for Energy (LIME), 00133 Roma, Italy; suhas.chandrasekaran@students.uniroma2.eu (S.C.); emanuela.sgreccia@uniroma2.it (E.S.); 3 Institute for the Study of Nanostructured Materials, ISMN-CNR, Monterotondo Stazione, 00015 Roma, Italy; saulius.kaciulis@cnr.it; 4 Aix Marseille University, CNRS, MADIREL (UMR 7246) and International Laboratory: Ionomer Materials for Energy (LIME), Campus St Jérôme, 13013 Marseille, France; suanto.syahputra@etu.univ-amu.fr (S.S.); florence.vacandio@univ-amu.fr (F.V.); philippe.knauth@univ-amu.fr (P.K.)

**Keywords:** hydrothermal carbonization, biowaste, electrocatalysis, ORR, CO_2_RR

## Abstract

Hydrothermal carbonization (HTC) serves as a sustainable method to transform pine needle waste into nitrogen-doped (N-doped) hydrochars. The primary focus is on evaluating these hydrochars as catalytic electrodes for the oxygen reduction reaction (ORR) and carbon dioxide reduction reaction (CO_2_RR), which are pivotal processes with significant environmental implications. Hydrochars were synthesized by varying the parameters such as nitrogen loading, temperature, and residence time. These materials were then thoroughly characterized using diverse analytical techniques, including elemental analysis, density measurements, BET surface area analysis, and spectroscopies like Raman, FTIR, and XPS, along with optical and scanning electron microscopies. The subsequent electrochemical assessment involved preparing electrocatalytic inks by combining hydrochars with an anion exchange ionomer (AEI) to leverage their synergistic effects. To the best of our knowledge, there are no previous reports on catalytic electrodes that simultaneously incorporate both a hydrochar and AEI. Evaluation metrics such as current densities, onset and half-wave potentials, and Koutecky–Levich and Tafel plots provided insights into their electrocatalytic performances. Notably, hydrochars synthesized at 230 °C exhibited an onset potential of 0.92 V vs. RHE, marking the highest reported value for a hydrochar. They also facilitated the exchange of four electrons at 0.26 V vs. RHE in the ORR. Additionally, the CO_2_RR yielded valuable C_2_ products like acetaldehyde and acetate. These findings highlight the remarkable electrocatalytic activity of the optimized hydrochars, which could be attributed, at least in part, to their optimal porosity.

## 1. Introduction

The increasing interest in biomass-to-char conversion stems from a confluence of factors: economic incentives, sustainability imperatives, and a rising demand [1,2]. The transformation of lignocellulosic biomass waste into char is particularly appealing in addressing solid waste management, reducing raw material costs, and offering tailor-made properties for diverse applications. Bio-based char, with its carbon-rich composition, exhibits distinctive chemical, physical, and biological characteristics, depending on the carbonization technologies used in its production [3]. It is therefore an excellent candidate for various applications, including soil quality improvement, environmental remediation, enzyme immobilization, and sensing [4]. New materials derived from bio-based chars are also gaining attention for catalytic applications, as recently reviewed by Lyu et al. [5], Lee et al. [6], Xiong et al. [7], and Cao et al. [8] amongst others.

For most of the current renewable energies, even if well established, there is a need to mitigate the production costs and minimize the use of raw materials. In this context, bio-based char has demonstrated potential applications in renewable energy conversion and storage, as recently reported, particularly in microbial fuel cells, supercapacitors, and batteries (metal–ion and metal–air) [9,10,11]. In recent years, there has been notable interest in the utilization of hydrothermal carbonization (HTC) for converting biomass waste into valuable carbonaceous materials [12,13,14,15,16,17,18,19,20]. This method has garnered attention due to its capability to produce hydrochars (HCs) with desirable characteristics that enhance their efficient utilization across various applications, particularly in the materials domain [21,22,23,24]. Hydrothermal carbonization is an eco-friendly thermochemical conversion method utilizing subcritical water to convert wet or dry biomass into carbonaceous products by fractionating the feedstock. The carbonization temperature is moderate, typically in the range of 180–300 °C [17] and depends on the starting materials and their decomposition temperatures. This process effectively hydrolyzes and dehydrates biomass, resulting in hydrochars with a substantial content of oxygenated functional groups, contributing to a richer surface chemistry compared to pyrochars [3]. Moreover, HTC has the capability to leach inorganic elements into the liquid phase, reducing the ash content [25]. While significant advancements have been made, a hydrochar is a relatively new type of bio-based char compared to established options like pyrochars. This means that there is a smaller pool of existing research and a less complete understanding of its properties and applications, necessitating further research to unlock its full potential.

N-doping of nitrogen-poor feedstocks introduces N-containing functional groups into the carbon framework from an external source. These nitrogen species may influence the electrochemical properties, the surface chemistry, the surface charge, and the surface wettability of the hydrochar, which can further influence the catalytic performance [26]. Different methods have been employed to introduce nitrogen into a carbon framework via thermochemical processes [27,28,29,30]. Notably, it has been demonstrated that nitrogen-containing compounds can deeply penetrate the biomass structure under HTC conditions [18]. This phenomenon facilitates the increased nitrogen integration within the bulk of the resulting hydrochar, thereby tuning the potential impacts on the electrochemical properties. Currently, the exploration of heteroatom-doped carbon is in its early stages, underscoring the need for further dedicated efforts for guiding the future endeavours in material design.

In this context, we investigated the catalytic properties of nitrogen (N)-doped hydrochars derived from the HTC of pine needle waste, for two electrochemical reactions with environmental relevance: oxygen reduction (ORR) and carbon dioxide reduction (CO_2_RR). To the best of our knowledge, the utilization of hydrochars for the fabrication of electrodes for these reactions is poor in the existing literature [31,32,33,34]. On the other hand, strategies enhancing the greenness of electrode materials are crucial for developing materials planned with a ‘benign by design’ approach.

In basic conditions, the ORR has a faster kinetic and was demonstrated to be feasible on various carbon-based electrocatalysts without the addition of critical raw materials [35,36,37]. Some papers report the use of biomass-derived carbons as electrocatalysts [38,39,40,41,42] pointing out the importance of the selection of biomass resources and synthesis procedures [43].

The CO_2_RR is a process of considerable interest due to its capacity to address the environmental concerns related to CO_2_ emissions and to contribute to the development of greenhouse gas (GHG) neutral sustainable energy systems. An added value is the conversion of CO_2_ into valuable products including hydrocarbons, formic acid, and multi-carbon products [32]. In all cases, the presence of anion exchange ionomers (AEIs) in the catalyst layers, required for the hydroxide ion transport, is paramount for high electrocatalytic performance [44].

The adoption of the HTC process could enable the fabrication of ORR and CO_2_RR electrocatalysts with much lower environmental impact showing comparable performance as the state-of-the-art materials.

Here, we present a study that addresses a current knowledge gap in hydrochar utilization for ORR and CO_2_RR, thereby enhancing the understanding and application of this promising material in electrocatalysis. This involves the preparation of N-doped hydrochars at moderate temperatures, thorough characterization, and an in-depth exploration of their electrocatalytic prowess for both ORR and CO_2_RR. Significantly, novel electrocatalytic inks have been formulated by combining hydrochars with an anion exchange ionomer (AEI) to harness their synergistic effects. To the best of our knowledge, there are no existing literature reports on catalytic electrodes that simultaneously integrate both a hydrochar and AEI.

## 2. Results and Discussion

### 2.1. Synthesis and Characterization of Hydrochars

Table 1 summarizes the experimental conditions for the carbonization of pine needles using urea as the N-dopant. In addition, the yield, density (δ), and elemental composition of the resulting hydrochars are reported.

**Figure 1:**
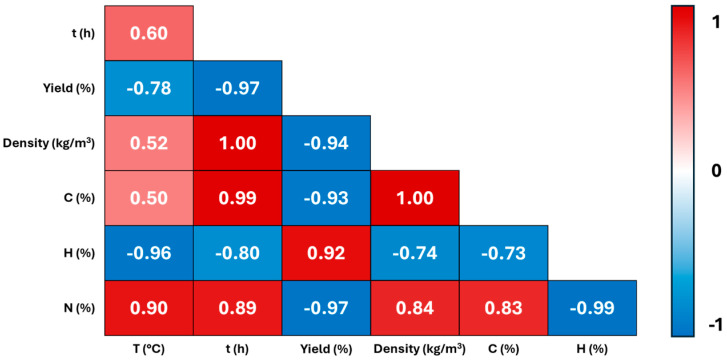
Correlogram of HTC temperature (°C), time (h), HC yield, density, C (%); H (%); N (%).

The correlogram (Figure 1), produced by using the Minitab^®^ version 21.4 statistical software, provides a visual representation of the relationships within the dataset.

The positive correlations are visualized in red, indicating the variables that change together in the same direction, while the negative correlations are represented in blue, suggesting the variables that vary inversely. The numerical values in the correlogram reflect the correlation coefficients for all the variable pairs, with the colour intensity indicating the strength of the correlation. There is a robust negative correlation between yield and density. Higher HTC temperatures and longer reaction times lead to decreased yields and higher apparent densities. Time emerges as a more significant parameter influencing yield and density, compared to temperature, under the investigated HTC conditions.

Examining the influence of the HTC temperature and time on the elemental composition of hydrochars reveals a highly significant correlation with the N content. The data provided in Table 1 report, indeed, that the nitrogen percentage (N%) increased from 2.50% to 4.45% as the temperature increased from 230 °C for 1 h to 260 °C for 6 h. This is likely due to the intensified thermal decomposition processes occurring at higher temperatures and longer durations. Under these conditions, chemical bonds break more extensively, releasing gases, including nitrogen-containing compounds. This phenomenon contributes to the observed increase in nitrogen content (nitrogen doping) in the resulting carbonaceous products. A highly robust positive correlation between the HTC time and carbon (C) content is observed. The longer reaction times allow for extended decomposition and carbonization processes, facilitating the greater expulsion of volatile components and resulting in a higher carbon content. Although less pronounced, a positive correlation is also evident between the C content and HTC temperature. Conversely, a highly significant negative correlation is observed between both the temperature (T) and time (t) and hydrogen content, with temperature exerting a greater influence. These observations align well with the trends observed in regard to density and yield for the produced carbonaceous structures (see above).

**Figure 2:**
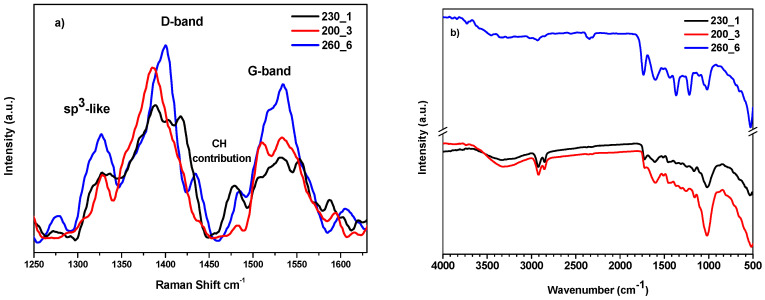
(**a**) Raman and (**b**) FTIR spectra of the samples.

The Raman and the FTIR spectra of the samples are reported in Figure 2. The various carbon materials obtained from hydrochars depend greatly on the ratio between the sp^2^ (graphite-like) and sp^3^ (diamond-like) regions. The Raman spectra (Figure 2a) of the samples reflect this dependency and appear very complex showing the typical features of hydrogenated amorphous carbon with graphitic carbon domains [45,46].

The D and G bands can be identified in the spectra. The D band is related to the presence of defects or disorder in the graphite lattice. The G band is associated with the in-plane vibrational motion of the sp^2^ hybridized carbon atoms in the hexagonal lattice of graphite [47]. Many factors can contribute to the broadening of the signals: (i) the size of carbon clusters: larger clusters may exhibit different vibrational properties compared to smaller ones; (ii) the cluster distribution: variations in the distribution of carbon clusters within the material; (iii) the nature of chemical bonding: changes in bonding configurations, such as the presence of functional groups or impurities. However, it has been observed that in chars obtained at relatively low temperatures and not aged, the D and G bands do not represent graphitic structures or defects but rather polyaromatic rings of different sizes. In particular, the D band at around 1380–1400 cm^−1^ could be related to fused aromatic rings and the G band at around 1520–1550 cm^−1^ could be assigned to aromatic ring breathing [48]. This statement seems to be confirmed by the XPS results. The position and relative shift in the D and G bands in Raman spectra can provide information about the structural characteristics of hydrochars. It is mentioned that the shift in these bands is associated with the degree of aromatization, and this is influenced by the temperature of formation [49], although there is controversy regarding the correlation of changes in the D and G bands with the char structure [50]. Comparing the three spectra in Figure 2a, the sample treated at higher temperatures for longer times (260_6) presents a maximum shift in D and G bands and a higher intensity for the G band, indicating an increase in the number or size of crystallites.

Other peaks are present in the Raman spectra: signals centred at 1325 cm^−1^ are the result of the vibration of sp^3^ carbons, while at around 1470–1490 cm^−1^, there is the contribution of C–H vibrations originated from single or fused aromatic rings [45,51].

The FTIR analysis is shown in Figure 2b. The spectra of samples 200_3 and 230_1 seem very similar, while the spectrum of 260_6 shows a different pattern. The hydroxyl band appears at 3000 cm^−1^ and can provide information about the water content of the different hydrochars, indicating the hygroscopic nature of the material. The intensity of the band is reduced with increasing HTC temperature, higher for samples 200_3 and 230_1, lower for 260_6. Aliphatic C-H stretching vibrations (around 2930 and 2850 cm^−1^), indicating sp^3^ carbons, are more pronounced for the samples treated at a low temperature. All the samples show the presence of carboxylic/carbonylic groups at 1735 cm^−1^, but the peak is more intense for samples 230_1 and 260_6; other peaks are present at 1608 cm^−1^ corresponding to aromatic C=C stretching. An aromatic C=C ring structure and CH_3_ deformations are at 1420 and 1370 cm^−1^, respectively. The sample 260_6 shows a band at 1223 cm^−1^, slightly visible for the sample 230_1, corresponding to aromatic C-O stretching. Aliphatic ether C–O and alcohol C–O at 1021 cm^−1^ appear stronger for the sample 230_1 and 200_3 [52,53].

The highly graphitic sample 260_6 has the highest water contact angle (129°, SI, Figure S1), in agreement with the low intensity of the O-H band in the FTIR spectrum, whereas the intermediate value belongs to the sample 230_1 (97°). The lowest value (89°) corresponds to a lower degree of graphitization (200_3) and indicates a higher content of hydrophilic oxygenated surface groups.

**Figure 3:**
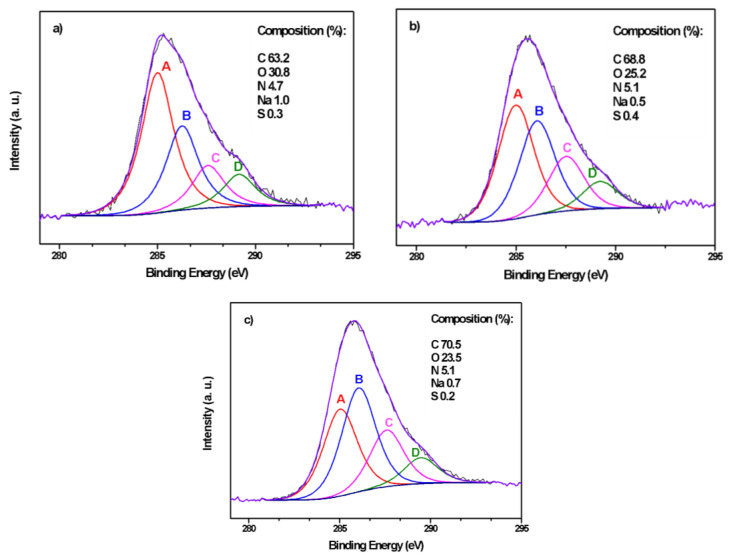
XPS spectra of the C 1s region for the samples (**a**) 200_3; (**b**) 230_1; (**c**) 260_6.

In Figure 3 are reported the XPS spectra of the C 1s region for all the samples. The deconvolution of the C 1s signal into four synthetic peaks showed that carbon is mostly present as aliphatic and aromatic carbon (component A, red line) and alcoholic or ether (furans, pyrans, etc.) carbon (component B, blue line) [54]. The other signals are due to ketone and C-N bonds (component C, pink line) and carboxylic carbon (component D, green line).

The intensity distribution of the four components varies with the type of sample as reported in Figure 3. Nitrogen is always present in pyrrolic/aminic/amidic bonds (400.2–400.6 eV) [55,56]. The O 1s spectra, reported in the SI (Figures S2–S4), confirm these assignments.

Comparing the composition reported via XPS with the results of the elemental analysis, it is possible to note that via XPS the carbon/oxygen ratio increases in the order 200_3, 230_1, 260_6, while via elemental analysis, the minimum value is for the 230_1 sample, indicating a greater content of oxygenated groups. In general, in the XPS analysis, the carbon content is higher with respect to the elemental analysis. Considering that XPS is a surface technique, the results suggest a higher concentration of carbon on the surface, in agreement with previous observations [57,58,59].

**Figure 4:**
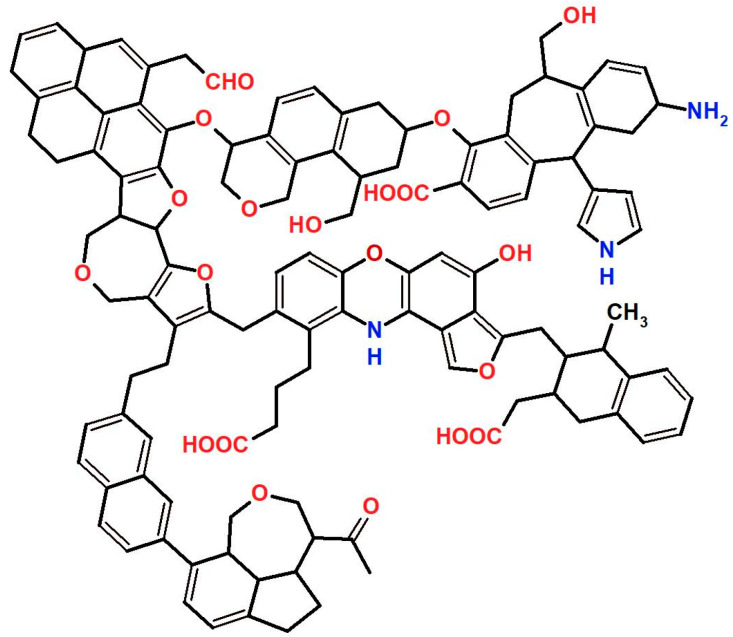
Schematic structure of hydrochar. In red, the oxygenated functionalities; in blue, the nitrogen functionalities.

A highly simplified structure of a hydrochar is proposed in Figure 4.

The BET isotherms of the sample 200_3 are characteristic of a macroporous material without micropores; the BET surface area (4.7 m^2^/g) is typical of non-activated hydrochars. The correlation between the porosity and density was analyzed by Brewer et al. [60]. As reported previously, the porosity of a hydrochar increases until 230 °C, while a higher temperature has a negative response [61]. The densities reported in Table 1 are consistent, with the lowest density observed for the sample 230_1.

The optical micrographs (Figure S5, SI) show that the hydrochar electrodes 200_3 and 260_6 are quite dense, whereas the electrode 230_1 presents a more porous and open microstructure. The hydrochar component is clearly visible on top of the fibrous structure of the carbon paper substrate. The SEM micrographs (Figure S6, SI) confirm this observation.

### 2.2. Hydrochar Catalytic Electrodes for ORR and CO_2_RR

A typical cyclovoltammetric determination of a hydrochar electrode’s capacitance in the non-Faradaic region is shown in SI (Figure S7a). The capacitances, obtained from a linear fit of the current vs. scan rate according to the equation, j = C(dU/dt) (Figure S7b), are reported for all the samples in SI (Table S2). These data can be compared with values obtained via impedance spectroscopy. Figure S8 (SI) presents impedance spectra for all the samples determined under the same conditions. The best-fit values of the various equivalent circuit elements are also reported in the SI (Table S2). The electrode 230_1, made using a hydrochar synthesized at an intermediate HTC temperature, has clearly the highest capacitance as corroborated via impedance spectroscopy, with the highest value of the circuit element representing the electrode capacitance (Table S2). This sample presents, thus, the highest electrochemically active surface area (ECSA), which is consistent with the optical and SEM micrographs showing that the layer of sample 230_1 is the most dispersed among the hydrochar electrodes.

**Figure 5:**
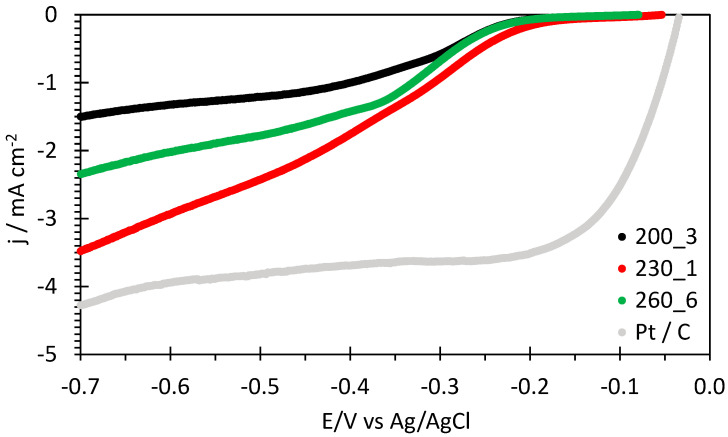
Comparison of linear sweep voltammograms for the oxygen reduction reaction of hydrochar electrodes and a benchmark Pt/C cloth electrode at 1500 rpm RDE speed in oxygen-saturated 0.1 M KOH solution.

A comparison of linear sweep voltammograms for the oxygen reduction reaction of the hydrochar samples is represented in Figure 5 together with a benchmark Pt/C cloth electrode.

**Figure 6:**
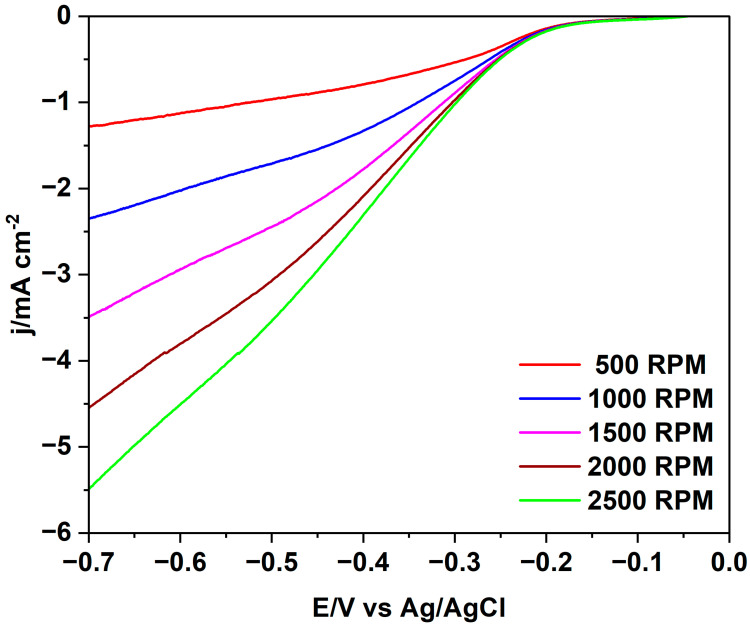
Linear sweep voltammograms of a 230_1 hydrochar electrode for the ORR in oxygen-saturated 0.1 M KOH at various RDE speeds.

The sample 230_1 has clearly the highest electrocatalytic activity for the ORR, and quite large current densities are attained at 1500 rpm. Linear sweep voltammograms at various RDE speeds are reported in Figure 6. Similar figures for the other hydrochar electrodes are shown in the SI (Figure S9).

Table 2 presents the various electrochemical parameters of the hydrochar electrodes for the ORR in alkaline solution. The potentials vs. the reversible hydrogen electrode (RHE) are calculated from the potential vs. Ag/AgCl according to Equation (1):

E(vs. RHE/V) = E(vs. Ag/AgCl) + 0.197 + 13 × 0.059(1)

The highest onset and half-wave potentials reported in Table 2 are observed for sample 230_1, indicating the best electrocatalytic properties. This onset potential is to our knowledge the highest value reported in the literature for hydrochars (Table 3). The value of 0.9 V vs. RHE reported in [34] refers to a hydrochar treated at 800 °C with a higher energy consumption.

**Figure 7:**
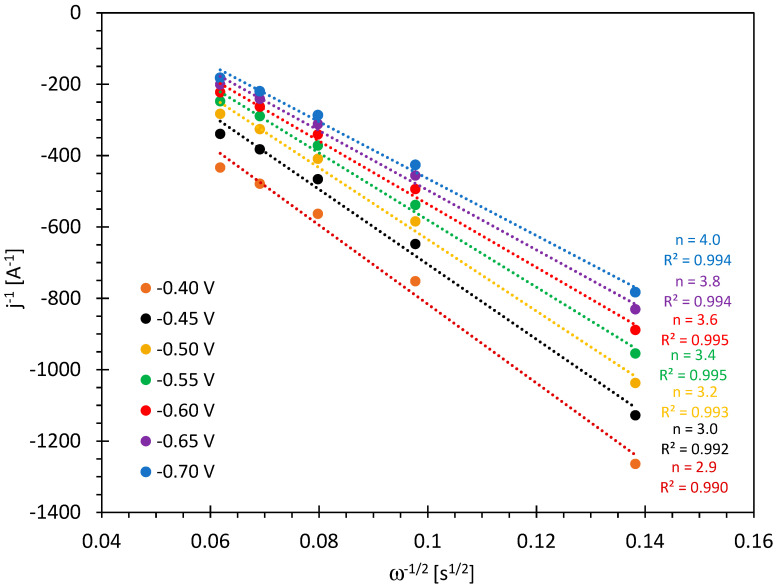
Koutecky–Levich plots for the electrode 230_1 at 1500 rpm RDE speed in oxygen-saturated 0.1 M KOH for different electrode potentials (E/V vs. Ag/AgCl). The number of exchanged electrons is indicated for each straight line.

The number of exchanged electrons can be obtained from Koutecky–Levich plots. The plots for the hydrochar electrode 230_1 at various overpotentials are reported in Figure 7. The corresponding figures for the other samples can be found in the supporting information (Figure S10). The number of exchanged electrons increases with the overpotential and reaches four at the highest cathodic potential.

The numbers shown in Table 2 at 0.26 V vs. RHE indicate that the hydrochar 200_3 gives mostly two-electron reduction, whereas the samples 230_1 and 260_6 are able to provide a substantial amount of four-electron reduction.

Finally, the Tafel plots reported in the SI (Figure S11) give slopes indicating two-electron transfer as a rate-determining step; the lower slope for the sample 230_1 (Table 2) is consistent with the better electrocatalytic activity. Altogether, there is a clearly better performance of this sample that can be attributed to a highly electrochemically active surface area, related to the optimal hydrothermal treatment conditions.

**Figure 8:**
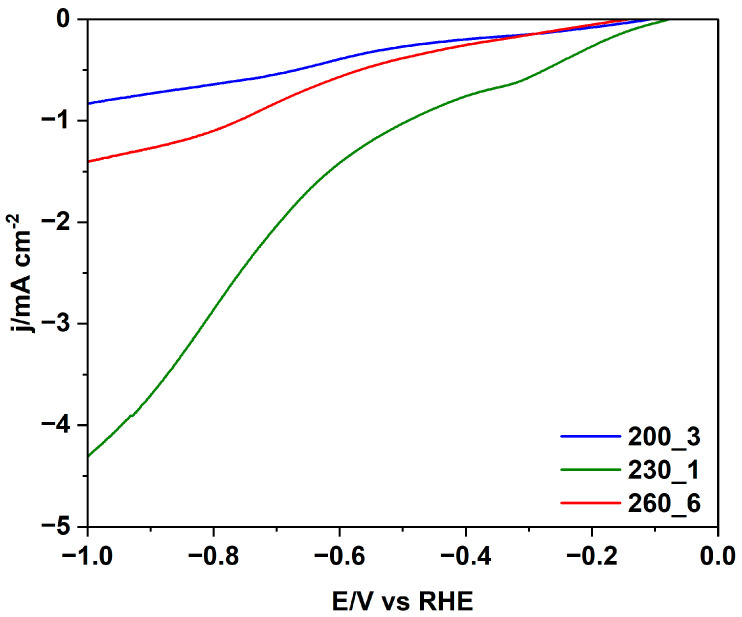
Comparison of linear sweep voltammograms of hydrochar electrodes for the CO_2_RR in CO_2_-saturated 0.1 M KHCO_3_ at 2500 rpm RDE speed.

This advantage is also very clearly observed for the CO_2_ reduction reaction (CO_2_RR). The comparison of the hydrochar electrodes is shown in Figure 8. There is again a large advantage for sample 230_1 and, as for the ORR, the sample 200_3 presents the lowest performance.

**Figure 9:**
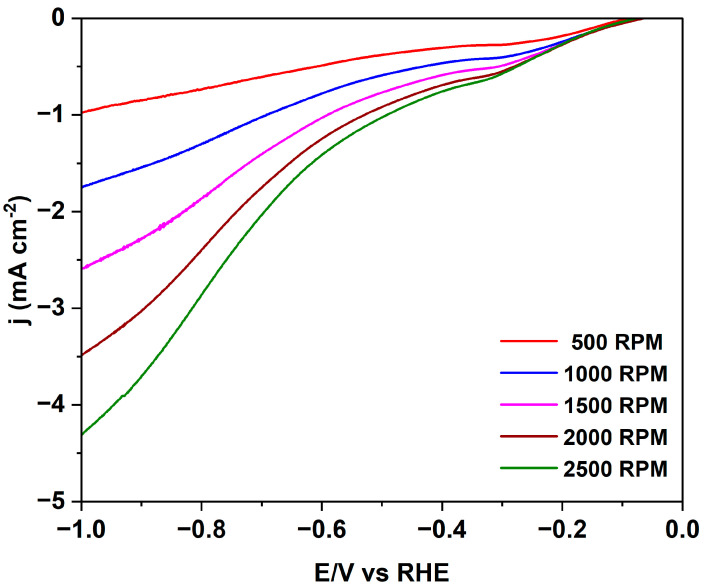
Linear sweep voltammograms of sample 230_1 for the CO_2_RR in CO_2_-saturated 0.1 M KHCO_3_ at various RDE speeds.

The linear sweep voltammograms at various RDE speeds are shown for hydrochar 230_1 in Figure 9.

Altogether, the LSVs confirm the superior electrocatalytic performance of 230_1 also for the CO_2_RR. Probably, the optimal porosity of this sample, related to an optimal HTC treatment, explains this result, because the order of current densities is the same as for the ORR, with a much higher current density observed for sample 230_1. CO_2_ is very sensitive to the presence of “basic” centres, and it is reported in the literature that the N-doping of carbons leads to an increase in the catalytic activity for the CO_2_RR. However, the hydrochar with the highest nitrogen content according to the elemental analysis (Table 1) does not show the best performance for the CO_2_RR; a similar result was reported recently by Fu et al. [42].

**Figure 10:**
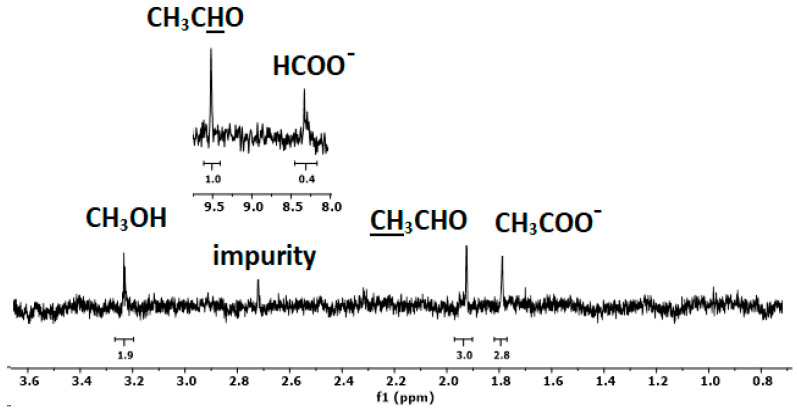
^1^H NMR spectrum of the solution after CO_2_RR with hydrochar sample 230_1.

NMR spectroscopy is a powerful investigative method for identifying the liquid products in CO_2_RR and has a low detection limit (<5 μM for methanol) [63]. The ^1^H NMR spectrum of sample 230_1 is reported in Figure 10. The spectrum was collected directly from the CO_2_RR solution using an external lock. In the high-field region, three peaks assigned to acetate, acetaldehyde, and methanol are present. The corresponding signal of acetaldehyde appears at a low field together with formate. Using the three hydrogens of acetaldehyde as an internal standard, the ratios between the products are 1:0.9:0.6:0.4 for acetaldehyde, acetate, methanol, and formate, respectively. In addition to the formation of methanol and formate, the CO_2_RR with these hydrochar electrodes leads thus to the generation of C_2_ products providing a means for producing valuable chemicals. The analysis of liquid products using HPLC and gaseous products using gas chromatography is the objective of future research.

By improving the microstructure and dispersion of the deposited hydrochar, one can significantly enhance the electrocatalytic activity (e.g., increase the number of exchanged electrons for the ORR), but also reduce the mass transport limitation of oxygen and CO_2_ to the active catalytic sites. The data reported here represent a significant improvement of the electrocatalytic performance of hydrochars in comparison with the previous literature, showing the great potential of these materials for electrocatalysis [33,64].

It is important to emphasize the significance of the presence of an excellent anion exchange ionomer (AEI) [44], specifically PPO-LC-TMA, which is crucial in designing high-performance electrodes.

## 3. Materials and Methods

### 3.1. Materials

Semi-dried pine needles were collected from an area surrounding the University of Perugia, Italy. The urea supplied by Merck KGaA (Darmstadt, Germany) was used as the nitrogen precursor. Other chemicals were of reagent grade and were used as received from Sigma-Aldrich. Carbon paper (AvCarb EP55) and Pt/C 60% cloth gas diffusion electrodes (0.5 mg/cm^2^) were purchased from the Fuel Cell Store.

### 3.2. Synthesis of Hydrochars

#### 3.2.1. Pine Needle Waste Pre-Treatment

Pine needles (PNs) were cut into 1–2 cm pieces and dried at 110 °C for 24 h. The resultant dried biomass was subsequently pulverized, sieved, and subjected to extraction using a toluene–methanol azeotrope in a Soxhlet apparatus for 8 h, achieving a 98% recovery rate. This extraction process aimed to eliminate the oil, waxes, and proteins from the biomass. Following the extraction, any residual solvent traces were efficiently removed under vacuum conditions at 80 °C. The pre-treated feedstock was then directly employed in the subsequent step.

#### 3.2.2. Hydrothermal Carbonization of Pre-Treated PNs with Urea

The synthesis of N-doped hydrochars was conducted using a 30 mL cylindrical pressure reactor fitted with a 13.0 kW/m^2^ heater and internal sensors for precise temperature control. Pre-treated PNs (2 g) were dispersed in 20 mL of water, supplemented with 0.025 g of sodium dodecyl sulfate (SDS) and 1.8 g of urea in a round-bottomed flask. The mixture underwent stirring at 60 °C and 700 rpm for 20 min before being loaded into the reactor. Subsequently, the reactor heater was activated and set to temperatures of 200 °C, 230 °C, and 260 °C, each for durations of 3, 1, and 6 h, respectively. The resulting hydrochar samples were designated as 200_3, 230_1, and 260_6. After the reaction time, the heater was deactivated, and the reactor was allowed to cool to room temperature, with the pressure reduced to the ambient level. Subsequently, product separation was achieved through filtration, and all the liquids were collected in a separate container. The hydrochar underwent drying in an oven at 105 °C for 24 h, followed by mortar and pestle grinding for 15 min and sieving (<0.3 mm), and finally, it was stored in an airtight container for subsequent analysis. The hydrochar yield was calculated using Equation (2):

Yield (%) = [weight of dried hydrochar (g)/weight of initial biomass (g)] × 100(2)

### 3.3. Catalytic Ink and Electrode Preparation

The anion exchange polymer used for the ink preparation was poly(2,6-dimethyl-1,4-phenylene oxide) (PPO) with N-pentyl-N,N,N-trimethylammonium side groups, called in the following PPO-LC (Long Chain)-TMA prepared in the LIME lab [65]. The inks for the catalytic layers were prepared from 40 mg of the hydrochar, 10 mg of PPO-LC-TMA (IEC = 0.953 meq/g) [44], and 500 μL of DMSO. The mixtures were left to undergo stirring overnight and then sonicated at RT for 30 min. An aliquot of the resulting pastes was uniformly spread over a layer of carbon paper [44]. The electrodes were vacuum dried at 40 °C for 4 h. The deposited mass was 0.25 ± 0.03 mg for all the samples, corresponding to a hydrochar catalyst loading of 0.7 mg/cm^2^.

### 3.4. Characterization

#### 3.4.1. Proximate Composition

The moisture (U), the volatile matter (VM), and the ash content were obtained according to EN ISO 18134-3 [66], EN 15148 [67], and EN 14775 [68], EN 51719 [69], respectively (Table S1, Supplementary Material). The fixed carbon (FC) was calculated using Equation (3):

FC (wt%) = 100 − VM (wt%) − U (wt%) − Ash (wt%)(3)

#### 3.4.2. Elemental Analysis

The elemental composition was determined using a UNICUBE^®^ elemental analyzer (Table 1 and Table S1, SI). The oxygen content was calculated using Equation (4):

O (%) = 100% − (C% + H% + N% + Ash%)(4)

#### 3.4.3. Bulk Density of Hydrochars

The bulk density was calculated according to the guidelines of the European Biochar Certificate [70], analogue VDLUFA-Method A 13.2.1 [71]. A dried, water-free sample of at least 300 mL was poured into a graduated cylinder, and its mass was measured. The sample’s volume was then recorded after it underwent ten compressions through falling. Subsequently, the bulk density (on a dry matter basis) in kg/m^3^ was calculated using the sample’s mass and volume.

#### 3.4.4. Brunauer–Emmett–Teller (BET) Analysis

The BET surface area was measured via nitrogen gas sorption at 77 K. The samples were vacuum degassed before the analysis.

#### 3.4.5. Water Contact Angle

The sessile drop method was applied using a Biolin Scientific Attension Theta Flex optical tensiometer. Samples of 3 µL of water were deposited at a 0.1 µL/s rate on the electrode surface. The drop shape and contact angle were analyzed according to the Young–Laplace equation, and an average of several experiments was determined.

#### 3.4.6. Microscopical Observations

The optical micrographs were produced using a Leitz Aristomet microscope. The Scanning Electron Micrographs (SEM) were obtained using a ZEISS Gemini SEM 500 at an acceleration voltage of 5 kV.

#### 3.4.7. Spectroscopic Analysis

^1^H-NMR spectra were recorded using a Bruker Avance 700 spectrometer operating at 700.18 MHz. After the CO_2_RR, the solution was collected and measured directly using an external look containing D_2_O. The Fourier Transform Infrared Spectroscopy (FTIR) spectra were obtained using a Perkin Elmer Spectrum 2 IR spectrometer equipped with an ATR ZnSi crystal. The Raman spectra were collected using a Optosky ATR 8300 Micro Raman spectrometer. The excitation wavelength was 785 nm and the laser power was 85 mW. X-Ray Photoelectron Spectra (XPS) were acquired by using an Escalab 250Xi (Thermo Fisher Scientific Ltd., Dartford, UK) with a monochromatic Al Kα (1486.6 eV) source. The powder samples were pressed on pure Au (99.99%) foil. The binding energy (BE) scale was corrected by positioning the C 1s peak of the aliphatic carbon at BE = 285.0 eV and controlling the position of the Fermi level at BE = 0 eV.

#### 3.4.8. Electrochemical Measurements

The three-electrode cell comprised a rotating disk electrode (RDE, 0.28 cm^2^ area, OrigaTrod, OrigaLys), a 4 cm^2^ Pt foil as a counter-electrode, and an Ag/AgCl reference electrode (E = 0.197 V vs. NHE). Oxygen-saturated 0.1 M KOH was used as the electrolyte for ORR, and CO_2_ saturated 0.1 M KHCO_3_ was used for CO_2_RR. Cyclic voltammetry (CV), linear sweep voltammetry (LSV), chronoamperometry (CA), and electrochemical impedance spectroscopy (EIS) measurements were performed at room temperature using a Biologic VMP3 potentiostat. The scan rates for the CV and the LSV measurements were 20–100 mV/s and 5 mV/s, respectively. The rotating speeds of the RDE were between 500 and 2500 rpm. The impedance spectra were recorded in a frequency range of 1 Hz–1 MHz (a.c. amplitude = 20 mV).

## 4. Conclusions

N-doped hydrochar-based catalytic materials were obtained via the hydrothermal carbonization of pine needle waste at moderate temperatures, a process recognized for its energy efficiency compared to the other carbonization methods.

Three different hydrochars were synthesized and mixed with an anion exchange ionomer to prepare an electrode ink used for the ORR and CO_2_RR. The electrocatalytic performances of the hydrochar synthesized at 230 °C are remarkable for the ORR, exhibiting a four-electron reduction capability and the highest reported value of the onset potential for a hydrochar. This sample exhibited an optimal porosity, and in CO_2_RR, various C_2_ products were obtained, including acetaldehyde and acetate, as determined by NMR spectroscopy. The addition of a high-performance hydroxide ion-conducting ionomer to the electrode ink was instrumental in achieving such high electrocatalytic performances.

Overall, the development of biomass-waste-derived hydrochar-based catalytic electrodes via low-energy hydrothermal carbonization represents a significant advancement in the field of sustainable catalysis. This represents the first report on catalytic electrodes simultaneously incorporating both a hydrochar and AEI.

## Figures and Tables

**Table 1:** HTC experimental conditions ^1^ for the synthesis of the different hydrochars and their corresponding yield, density, and elemental analysis.

Sample	T (°C)	Time (h)	Yield (%)	δ (kg/m^3^)	C (%)	H (%)	N (%)	O ^2^ (%)
200_3	200	3	60	224	49.72	5.49	2.34	37.85
230_1	230	1	61	136	45.70	5.31	2.50	39.51
260_6	260	6	55	320	53.68	4.75	4.45	31.79

^1^ pine needles: urea = 1:0.9 wt/wt ratio. ^2^ Calculated by difference.

**Table 2:** Electrochemical properties of hydrochar electrodes for the ORR in oxygen-saturated 0.1 M KOH. E_onset_ and E_1/2_ are the onset and half-wave potentials vs. RHE, n is the number of exchanged electrons for the ORR (at 0.26 V vs. RHE), and b is the Tafel slope.

Sample	E_onset ORR_ vs. RHE/V	E_1/2 ORR_ vs. RHE/V	n ORR	B ORR/mV
200_3	0.90	0.67	1.85	114
230_1	0.92	0.69	3.97	106
260_6	0.90	0.67	3.63	114

**Table 3:** Comparison of the onset potential (E_onset_) and number of exchanged electrons (n) of hydrochars from this work and from the literature.

Hydrochar (Synthesis Conditions)	Biomass Source	E_onset_ (V/RHE)	n	Ref.
230 °C 1 h (230_1)	pine needles	0.92	3.97 at 0.26 V/RHE	This work
200 °C 12 h, treated in KMnO_4_ and H_2_SO_4_ (pH~3)	extracted avocado seed	N/A	1.83 at 0.46 V/RHE	[33]
200 °C 12 h, treated in KMnO_4_ and NH_4_OH (pH~9)	extracted avocado seed	N/A	2.21 at 0.46 V/RHE	[33]
800 °C, N-doped	wood chips	0.90	~4 in the range 0.30–0.65 V/RHE	[34]
250 °C 12 h, activated in KOH and incorporation of MnO_2_	corncobs	0.73	3.27 at 0.21 V/RHE	[62]
250 °C 12 h, activated in KOH and incorporation of MnO_2_	coffee waste grounds	0.71	3.45 at 0.21 V/RHE	[63]
250 °C 12 h, activated in KOH and incorporation of MnO_2_	rice hull	0.61	2.84 at 0.21 V/RHE	[63]
250 °C 12 h, activated in KOH and incorporation of MnO_2_	coconut sawdust	0.60	3.23 at 0.21 V/RHE	[63]

## Data Availability

Data are contained within the article or Supplementary Materials.

## Supplementary Materials

The following supporting information can be downloaded at https://www.mdpi.com/article/10.3390/molecules29143286/s1: Elemental and proximate analyses, water contact angle measurements, XPS measurements, optical micrographs, and SEM images for hydrochars; plots and data concerning electrode capacitance; impedance spectra; voltammograms for ORR and CO_2_RR; Koutecky–Levich and Tafel plots.
