# Supplementary material for: Hydrochar from Pine Needles as a Green Alternative for Catalytic Electrodes in Energy Applications
Source: Molecules. 2024 Jul 11;29(14):3286. doi: 10.3390/molecules29143286 (PMC11278999; doi:10.3390/molecules29143286)
Supplement: Supplementary file 1 [file molecules-29-03286-s001.zip › molecules-3051728-supplementary.pdf]

Supporting Information

Hydrochar from pine needles as a green alternative for catalytic electrodes in energy applications

A. Marrocchi[1,*], E. Cerza[1], S. Chandrasekaran[2], E. Sgreccia[2], S. Kaciulis[3], L. Vaccaro[1], S. Syahputra[4], F. Vacandio[4], P. Knauth[4], M.L. Di Vona[2,*]

[1] *University of Perugia, Department of Chemistry, Biology and Biotechnology, via Elce di Sotto 8, 06123 Perugia, Italy*

[2] *Tor Vergata University of Rome, Dep. Industrial Engineering and International Laboratory: Ionomer Materials for Energy (LIME), 00133 Roma, Italy*

[3] *Institute for the Study of Nanostructured Materials, ISMN-CNR, 00015 Monterotondo Stazione, Roma, Italy*

[4] *Aix Marseille Univ, CNRS, MADIREL (UMR 7246) and International Laboratory: Ionomer Materials for Energy (LIME), Campus St Jérôme, 13013 Marseille, France*

Elemental and proximate analysis

Table S1. Elemental and proximate analysis of pine needles waste

**Elemental analysis [a] [wt. %]**				
C	H	N	S	O [c]
44.51	5.07	0.47	0.16	48.16
**Proximate analysis [a] [wt. %]**				
U [b]	VM	Ash	FC [c]	
10.26	84.89	1.63	3.21	

[a] Dry basis; [b] Air dry basis; [c] Calculated by difference

Water contact angle

a)

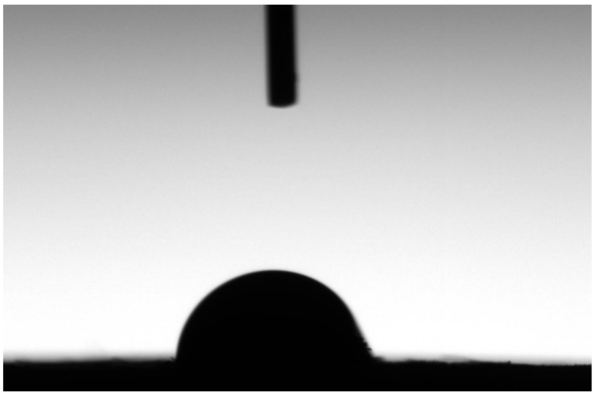

b)

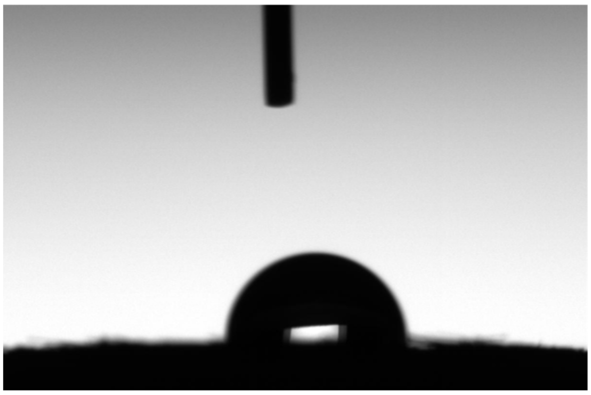

c)

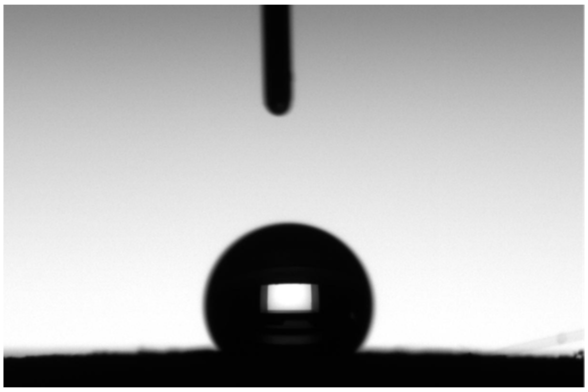

Figure S1. Water contact angle measurements for hydrochar samples: (**a**) 200_3 (ϴ = 89°), (**b**) 230_1 (ϴ = 97°), (**c**) 260_6 (ϴ = 129°).

XPS

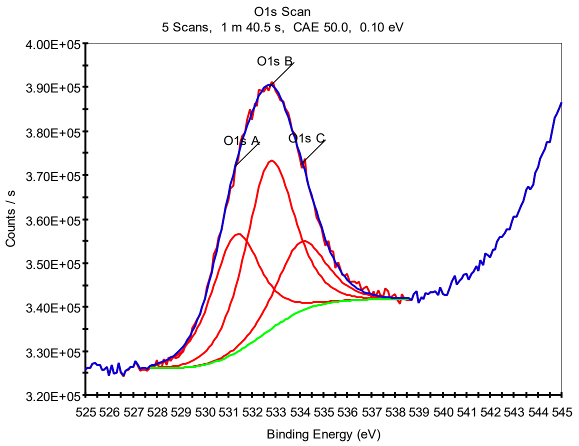

Figure S2. XPS of the O1s region for the sample 200_3. Component A at 531.3 eV –OH, C=O bonded to aromatic carbon; component B at 532.7 eV C-O bonded to aliphatic carbon; component C at 534.1 eV C-O carboxylic.

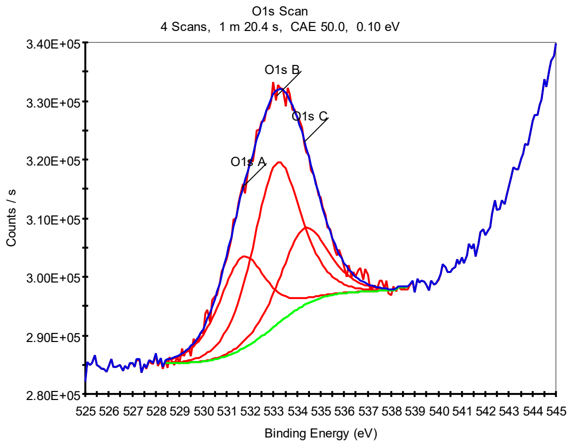

Figure S3. XPS of the O1s region for the sample 230_1. Component A at 531.7 eV –OH, C=O bonded to aromatic carbon; component B at 533.1 eV C-O bonded to aliphatic carbon; component C at 534.3 eV C-O carboxylic.

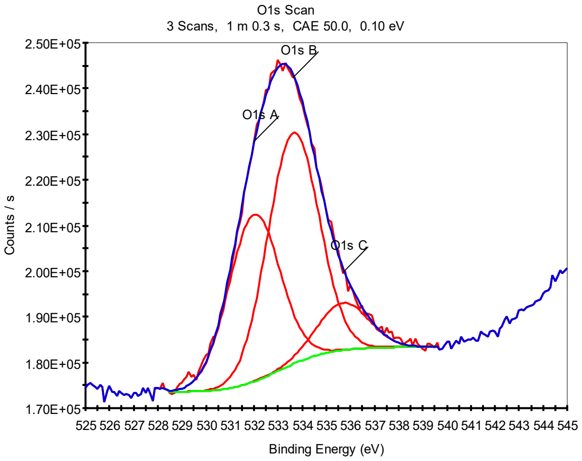

Figure S4. XPS of the O1s region for the sample 260_6. Component A at 532.0 eV –OH, C=O bonded to aromatic carbon; component B at 533.6 eV –C=O, C-O; component C at 535.7 eV –C-O, H_2O.

Optical Micrographs

a)

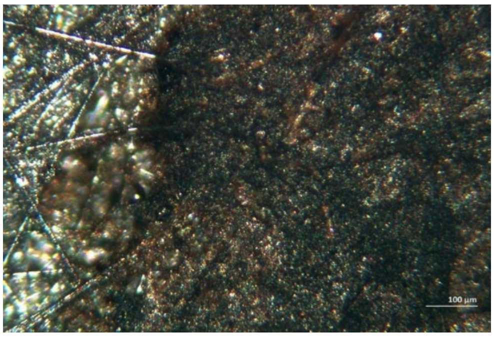

b)

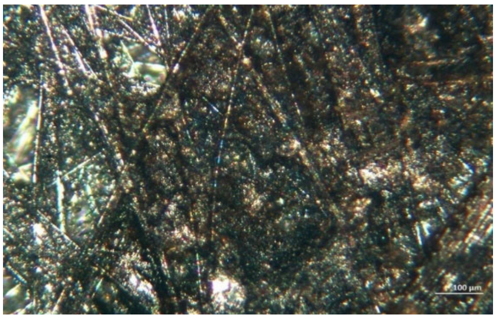

c)

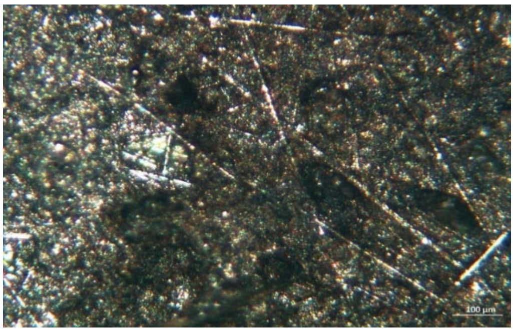

Figure S5. Optical micrographs of hydrochar samples: (**a**) 200_3, (**b**) 230_1, (**c**) 260_6.

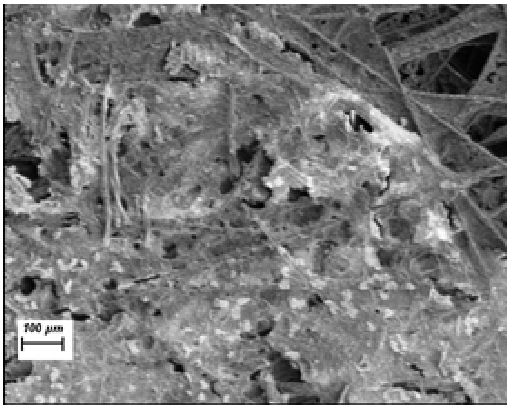

Figure S6. SEM image of hydrochar 230_1.

Electrode Capacitance

a)

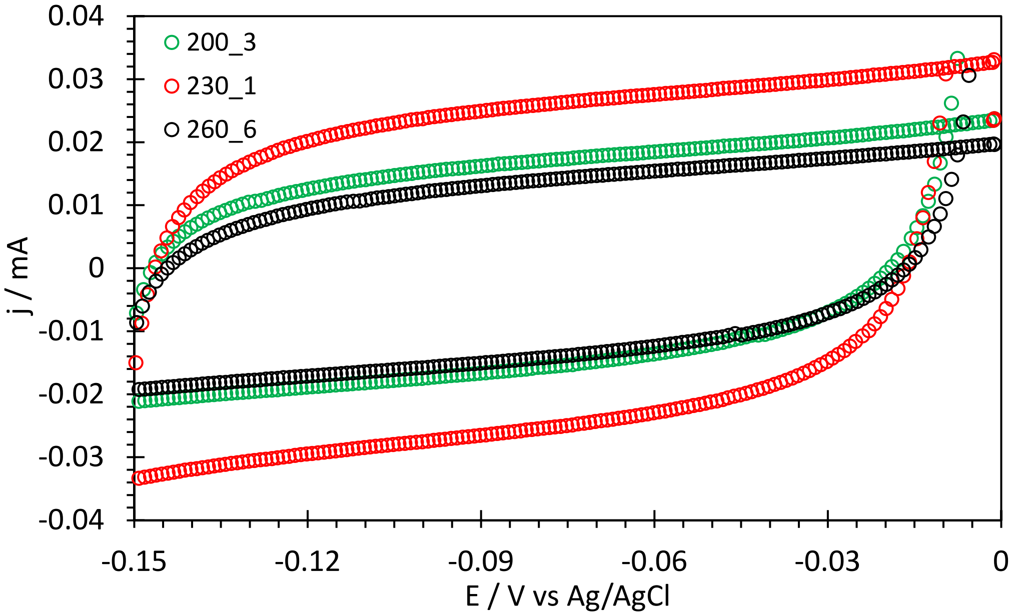

b)

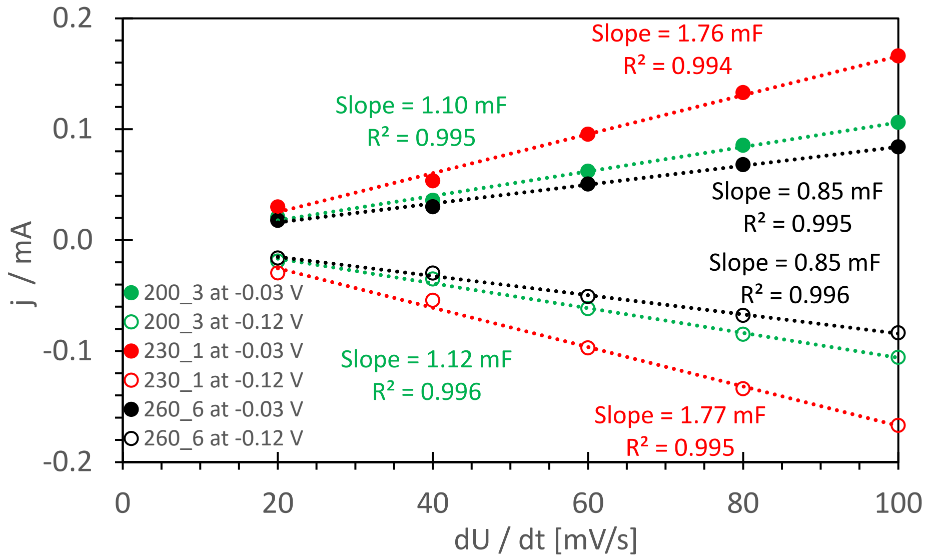

Figure S7. (**a**) Typical cyclovoltammograms in the non-Faradaic region of hydrochar electrodes in oxygen-saturated 0.1 M KOH solution at 20 mV/s. (**b**) Determination of the capacitance of electrodes at -0.03 V and -0.12 V vs Ag/AgCl at scan rates 20-100 mV/s according to equation 1.

The electrode capacitance *C* corresponds to the slope of the non-Faradaic current *j* as function of the potential scan rate *dU/dt*:

$$j = C \times dU/dt \quad \text{(S1)}$$

The capacitance values C of the hydrochar electrodes are reported in Table S2.

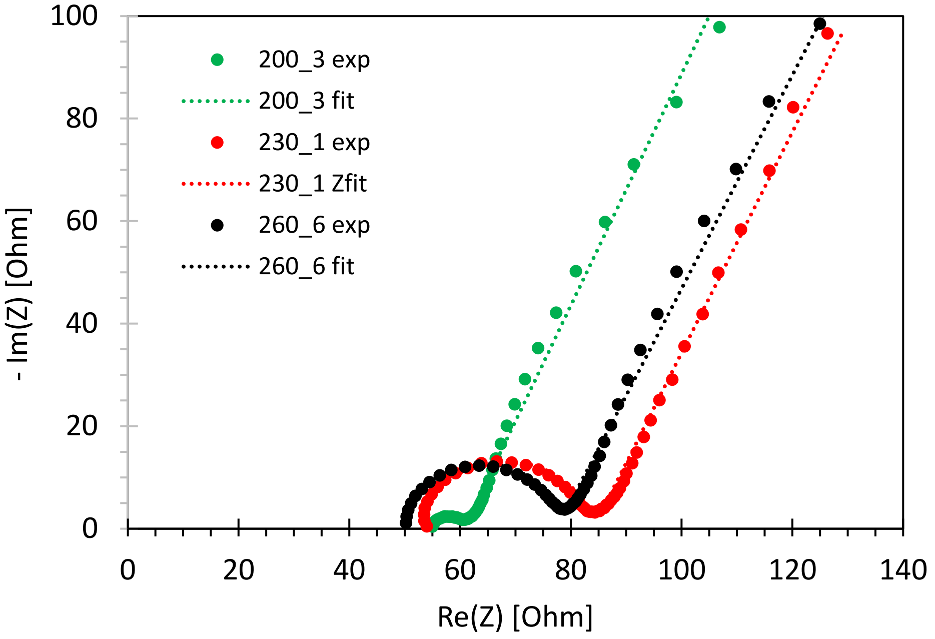

Figure S8. Impedance spectra of hydrochar electrodes in oxygen-saturated 0.1 M KOH solution.

The impedance spectra can be fitted by an equivalent circuit consisting of a resistance R1 (representing the electrolyte and electrode resistance), in series with a parallel arrangement of a resistance R2 (representing the charge transfer resistance) and a constant phase element Q2 (representing the interfacial capacitance) in series with a constant phase element Q3 (representing the electrode capacitance) that can be compared with dc capacitance data. The impedance of a constant phase element (CPE) can be written as follows:

$$Z(CPE) = \frac{1}{Q}(i\omega)^{-n} \quad \text{(S2)}$$

Q is the CPE value and n the CPE exponent; i is the imaginary unit and ω the angular frequency. $n = 1$ relates to an ideal capacitor and n = 0 to a pure resistor. n = 0.5 is indicative of a diffusion-limited process (Warburg impedance). The non-linear least square-fit data are reported in Table S2.

Table S2. D.c. electrode capacitance C and non-linear least-square fit impedance parameters for hydrochar electrodes.

Sample	C /μF	R1 /Ω	R2 /Ω	Q2 /μFs^{n-1}	n2	Q3 /μFs^{n-1}	n3
**200_3**	1110	54.9	5.8	8.4	0.87	1478	0.73
**230_1**	1765	52.1	32.0	1.3	0.87	2493	0.72
**260_6**	850	50.2	27.4	0.6	0.94	1111	0.72

Oxygen reduction reaction (ORR)

a)

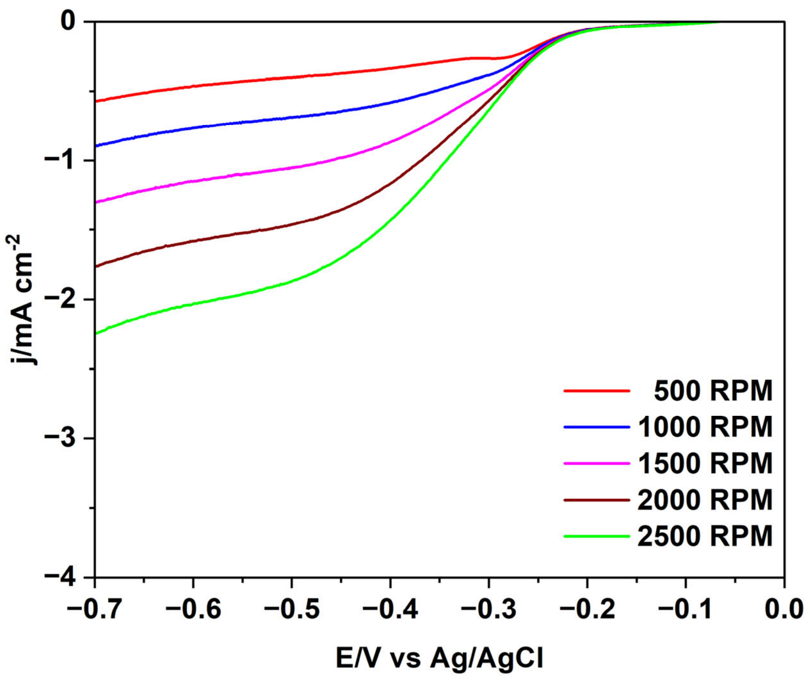

b)

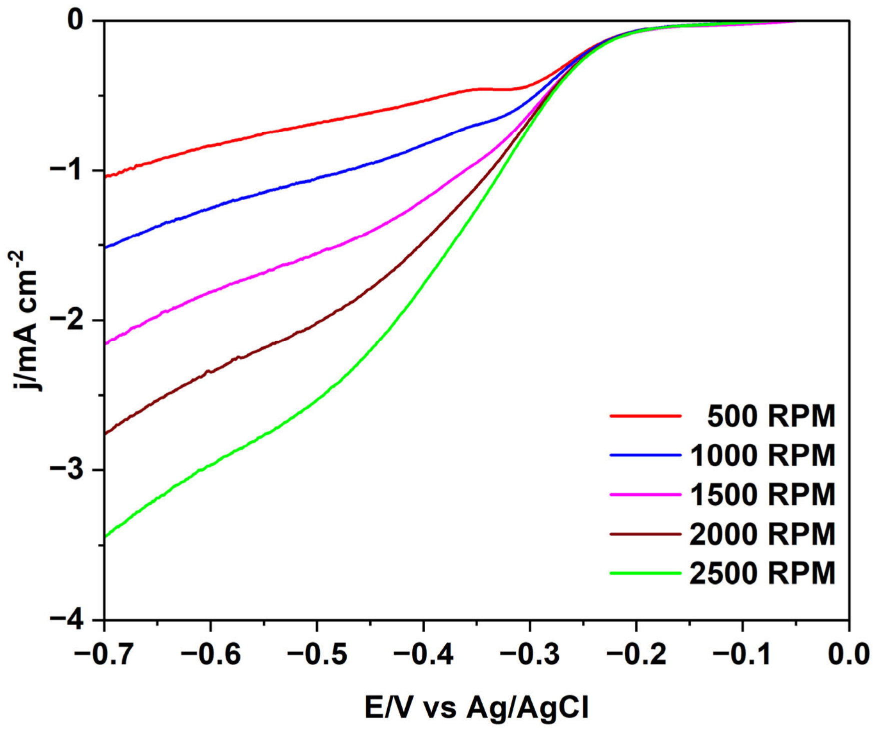

Figure S9. Linear sweep voltammograms for the ORR in oxygen-saturated 0.1 M KOH at various RDE speeds: (**a**) 200_3 and (**b**) 260_6 hydrochar electrodes.

The number of exchanged electrons in the ORR can be calculated using the Koutecký–Levich equation:

$$\frac{1}{i} = \frac{1}{i^k} + \frac{1}{B.\omega^{\frac{1}{2}}} \quad \text{(S3)}$$

i is the limiting current, i^k the kinetic current and ω the angular frequency of the RDE. B is the Levich slope that can be expressed by the equation:

$$B = 0.62\, A\, n\, F\, c(O_2)\, D(O_2)^{\frac{2}{3}}\, v^{\frac{1}{6}} \quad \text{(S4)}$$

A is the geometrical electrode area, *n* the number of exchanged electrons, *F* the Faraday constant (96485 C mol^{-1}). $c(O_2)$ the saturation concentration of oxygen (1.2 10^{-6} mol cm^{-3} in 0.1 M KOH), $D(O_2)$ the oxygen diffusion coefficient (1.9 x 10^{-5} cm^2 s^{-1} in 0.1 M KOH), and *v* the kinematic viscosity (8.7. 10^{-3} cm^2 s^{-1} in 0.1 M KOH). Koutecky-Levich plots for the hydrochar 230_1 electrode is reported at various overpotentials in Figure 7 of the main manuscript. The corresponding plots for samples 200_3 and 260_6 can be found in Figure S10.

a)

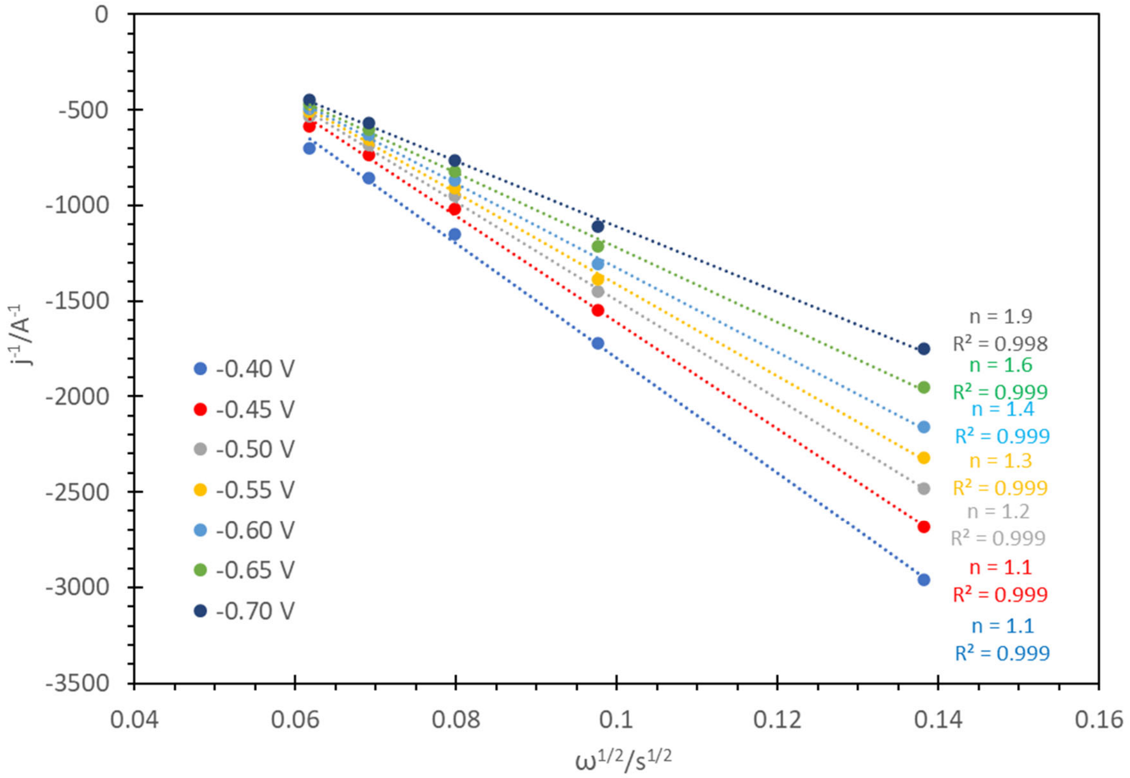

b)

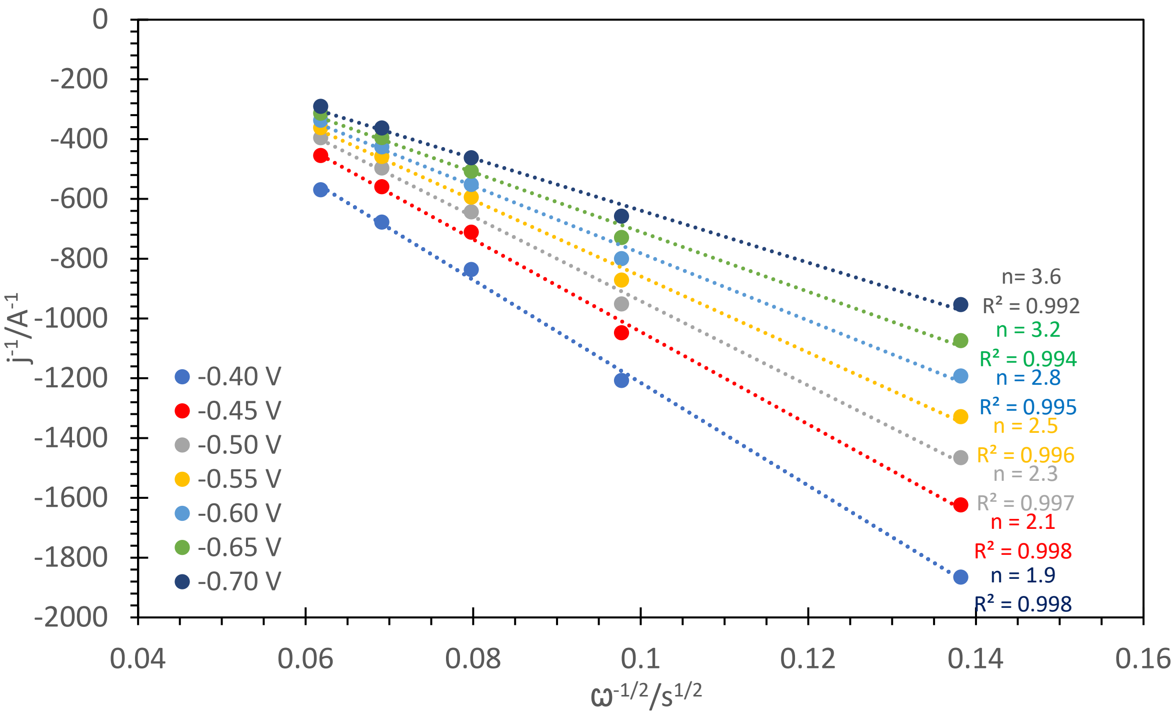

Figure S10. Koutecky-Levich plots at 1500 rpm RDE speed in oxygen-saturated 0.1 M KOH for different electrode potentials (E/V vs Ag/AgCl) for the samples (**a**) 200_3 and (**b**) 260_6. The number of exchanged electrons is indicated for each potential.

The Tafel equation between absolute values of the cathodic overpotential $|\eta|$ and the cathodic current density $|i|$ is:

$$|\eta| = \frac{2.3\,RT}{\alpha nF} \log\left(\frac{|i|}{i^{\circ}}\right) \qquad \text{(S5)}$$

R is the ideal gas constant, *T* the absolute temperature, α the symmetry coefficient, *n* the number of exchanged electrons, *F* the Faraday constant and i^o the exchange current density. Figure S11 shows Tafel plots at 1500 rpm for the hydrochar electrodes. A better electrocatalytic activity is generally attributed to a lower slope. The lowest slope of 106 mV/decade for sample 230_1 confirms its better electrocatalytic performance in comparison to other electrodes.

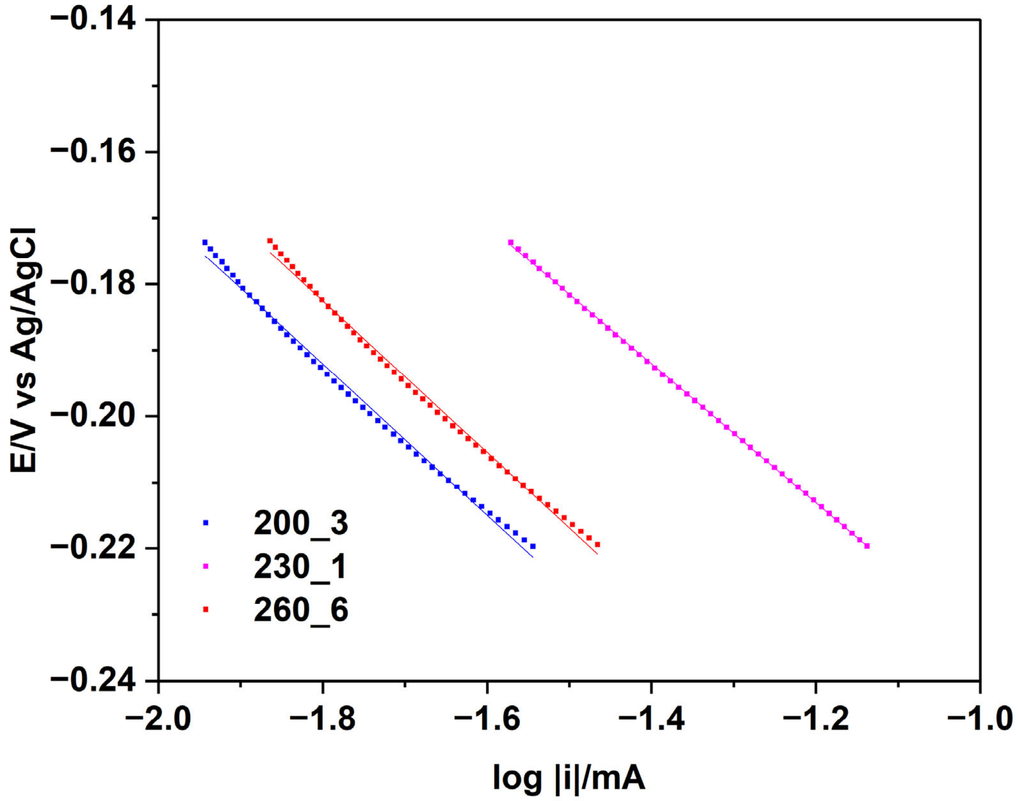

Figure S11. Comparison of Tafel plots of hydrochar electrodes for the ORR in oxygen-saturated 0.1 M KOH at 1500 rpm RDE speed.

CO_2 reduction reaction (CO_2RR)

a)

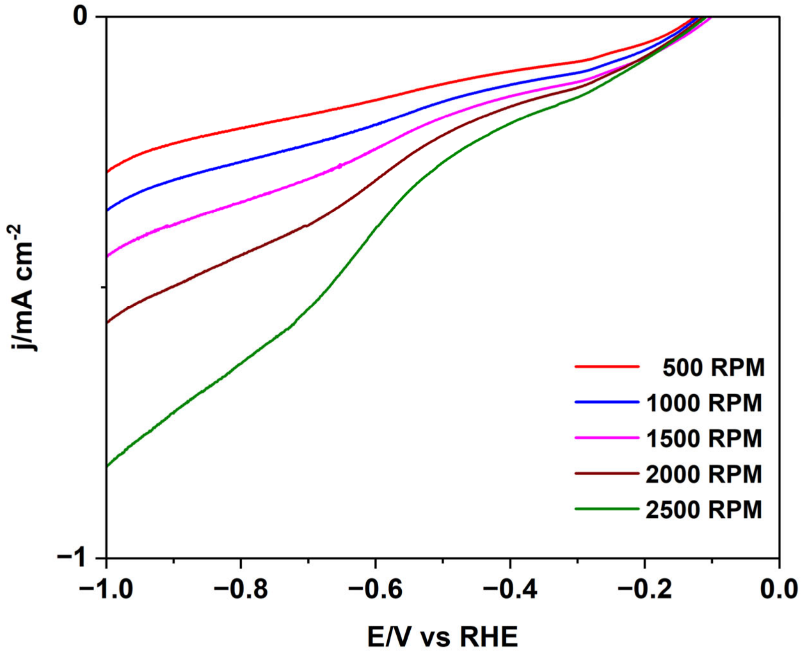

b)

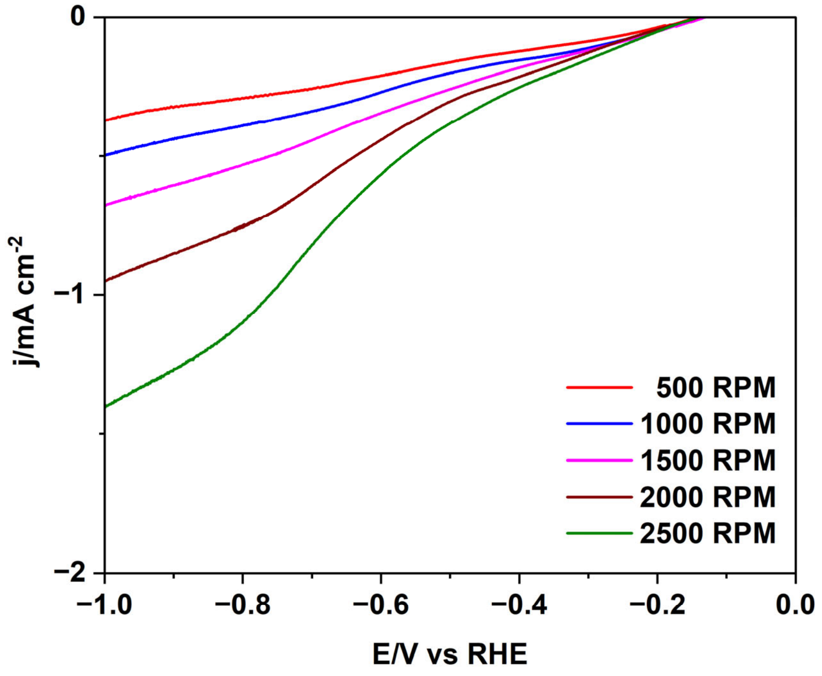

Figure S12. Linear sweep voltammograms for the CO_2RR in CO_2-saturated 0.1 M $KHCO_3$ at various RDE speeds: (**a**) 200_3 and (**b**) 260_6 hydrochar electrodes.
